# Reduced relapse in high risk acute myeloid leukemia and myelodysplastic neoplasms with permissive HLA-DPB1 mismatches and post-transplant cyclophosphamide

**DOI:** 10.1038/s41375-026-02907-4

**Published:** 2026-03-09

**Authors:** Portia Smallbone, Kai Cao, Rima M. Saliba, Yudith Carmarzzi, Gheath Al-Atrash, Michele Alvarez, Gabriela Rondon, Warren B. Fingrut, Yosra M. Aljawai, Amanda L. Olson, Amin M. Alousi, George L. Chen, Mark R. Tanner, Jun Zou, Jeremy L. Ramdial, Richard E. Champlin, Elizabeth J. Shpall, Betül Oran

**Affiliations:** 1 Department of Stem Cell Transplantation and Cellular Therapy, The University of Texas MD Anderson Cancer Center, Houston, TX USA; 2 Department of Laboratory Medicine, The University of Texas MD Anderson Cancer Center, Houston, TX USA

**Keywords:** Acute myeloid leukaemia, Myelodysplastic syndrome

## Abstract

Disease progression, graft-versus-host disease (GVHD), and non-relapse mortality (NRM) are the main causes of failure after allogeneic hematopoietic cell transplantation (SCT) for myeloid neoplasms. T-cell epitope–based models classify HLA-DPB1 mismatches by permissiveness and have been associated with differential risks of GVHD, relapse, and NRM. However, most studies were conducted before the routine use of post-transplant cyclophosphamide (PTCy) as GVHD prophylaxis for unrelated donor (UD) SCT. We retrospectively analyzed 541 adults undergoing 8/8 UD SCT with PTCy prophylaxis, categorized as DP-matched (DP-M, *n* = 176), permissive mismatch (DP-P, *n* = 219), non-permissive graft-versus-host (DP-NP-GVH, *n* = 82), or host-versus-graft (DP-NP-HVG, *n* = 64). Outcomes were compared across groups with stratification by disease risk and remission/MRD status. Two-year relapse incidence was lower with DP-P versus DP-M (18% vs 28%; HR 0.6, 95% CI 0.4–0.9, *p* = 0.03), most pronounced in high-risk AML/MDS, where relapse rates approached those of lower-risk disease. This effect persisted after adjustment for remission and MRD status. GVHD incidence was unaffected by DPB1 status. OS and PFS did not differ significantly; age and comorbidity were dominant predictors of NRM. In UD SCT with PTCy, DPB1-permissive mismatching reduces relapse in high-risk AML/MDS without increasing GVHD or NRM, supporting DP-P mismatching as an actionable donor-selection criterion.

## Introduction

Failure after allogeneic stem cell transplantation (allo-SCT) for myeloid malignancies is primarily driven by disease relapse, graft-versus-host disease (GVHD), and non-relapse mortality (NRM) [1, 2]. Donor selection strategies have been explored to improve post-transplant outcomes, with particular focus on the role of human leukocyte antigen (HLA)-DPB1 mismatching. Biological models based on T-cell epitope (TCE) groups suggest that permissive HLA-DPB1 mismatches are associated with a reduction in relapse risk after unrelated donor transplantation [3–5]. However, this benefit comes at the cost of a higher incidence of acute GVHD (aGVHD) [6]. More refined models, such as expression-integrated or TCE-Core approaches, suggest that the balance between the graft-versus-leukemia (GVL) benefit and GVHD risk depends not only by mismatch permissiveness, but also on expression context. Based on these findings and improved biological understanding, consensus guidelines have been updated to recommend selecting DPB1-matched or permissive-mismatched donors, while avoiding non-permissive mismatches [7].

Post-transplant cyclophosphamide (PTCy) has markedly improved outcomes in HLA mismatched transplants [8, 9]. Initially tested in haploidentical transplantation, PTCy-based GVHD prophylaxis has yielded outcomes comparable to those of fully matched transplants using conventional GVHD prophylaxis [10]. More recently, a phase III trial has shown that PTCy combined with a calcineurin inhibitor significantly improved GVHD-free, relapse-free survival (GRFS) in unrelated donor transplantation, reducing severe GVHD compared to standard prophylaxis [11]. The impact of HLA mismatching in the PTCy era was further explored in a large European Bone Marrow Transplant (EBMT) study involving over 17,000 patients, including 1500 who received PTCy [6]. This analysis indicated that PTCy does not fully abrogate the effects of HLA-DPB1 mismatching; however, associations between DPB1 mismatch and reduced relapse were less clear in the PTCy-treated groups. In parallel, recent EBMT studies focusing specifically on AML and MDS have advanced understanding of HLA-DPB1 mismatching by incorporating immunopeptidome divergence, TCE permissiveness, and directionality in 10/10 matched unrelated transplantation. These analyses demonstrate that relapse protection associated with DPB1 mismatching is driven by permissive subsets and directionality, albeit with associated increased in acute GVHD [12]. Notably, these leukemia-focused cohorts largely predated routine use of PTCy, and were conducted in heterogeneous, registry-based populations, leaving uncertainty regarding the impact of DPB1 permissiveness in a homogenous cohort treated uniformly with contemporary PTCy-based GVHD prophylaxis.

Despite these advances, relapse remains the leading cause of failure after allo-SCT, particularly in patients with high-risk myeloid malignancies. Given the poor outcomes associated with post-transplant relapse, multiple strategies, including intensifying conditioning regimens [13–15], administrating post-transplant maintenance therapy [16, 17], and incorporating immune-based approaches [18, 19], have been investigated to improve disease control. Whether donor-recipient HLA-DPB1 mismatching in the context of PTCy-based GVHD prophylaxis can reduce relapse without causing excess morbidity and mortality remains unclear. In this study, we evaluated the impact of HLA-DPB1 mismatching on transplant outcomes after 8/8 matched unrelated donor allo-SCT with PTCy for myeloid malignancies, stratified by disease risk. Specifically, we investigated whether HLA-DPB1 matching criteria improved relapse risk without increasing GVHD, NRM, and overall mortality.

## Methods

### Patient selection

We performed a retrospective analysis of 541 adult patients (aged 18 or older) with myeloid malignancies who underwent allo-SCT from matched unrelated donors at MD Anderson Cancer Center between 2011 and 2024. GVHD prophylaxis consisted of tacrolimus with mycophenolate mofetil in combination with standard dosing of PTCy (50 mg/kg/day on days +3 and +4). At our institution, this platform has been routinely applied across unrelated donor transplants since 2017. Both myeloablative (MAC) and reduced intensity conditioning (RIC) regimens were included. We categorized regimens as reduced intensity conditioning: (1) IV busulfan in a dose calculated to target an area under the curve (AUC) of 4000 μmol/min ± 10% with fludarabine or (2) melphalan 100 to 140 mg/m^2^ as a single dose with fludarabine. Myeloablative conditioning regimens consisted of one of the following IV busulfan-containing regimens: (1) nonfractionated busulfan with pharmacokinetic dose adjustment to achieve a target of an average daily systemic exposure, represented by the AUC of 5000 to 6000 μmol/min ± 10%, in combination with fludarabine or (2) fractionated busulfan with a fixed dose of 80 mg/m^2^ delivered daily on days –13 and –12, followed by busulfan on days –6 to –3, and dosed based on pharmacokinetic studies to achieve a target AUC of 16,000 to 20,000 μmol/min for the course with fludarabine [13, 20]. Hematopoietic stem sources comprised either peripheral blood (PB) or bone marrow (BM). Tacrolimus duration is standardized, with wean occurring at day +180. This study was approved by Institutional Review Board at the University of Texas MD Anderson Cancer Center, and the study was conducted in accordance with the Declaration of Helsinki.

### HLA matching and risk classification

HLA-DPB1 matching was classified according to previously published criteria [21]. Donor-recipient pairs matched at 8/8 loci were further categorized as DP-matched (DP-M), DPB1 permissive mismatched (DP-P), DPB1 non permissive mismatch in the GVH direction (DP-NP-GVH), or DPB1 non-permissive mismatch at host-versus-graft (HvG) (DP-NP-HVG) direction (Supplementary Fig. 1).

Patients were stratified into risk categories using disease-specific criteria. For acute myeloid leukemia (AML), risk classification was based on the 2017 European LeukemiaNet (ELN) [22] guidelines, and for myelodysplastic neoplasm (MDS), the Revised International Prognostic Scoring (IPSS-R) was applied [23]. High-risk disease was defined as adverse risk per ELN or high/very high-risk per IPSS-R. All other patients were considered low/intermediate risk. Patients with myeloproliferative disorders (MPN), chronic myelomonocytic leukemia (CMML), or chronic myeloid leukemia (CML) were classified separately as MPN/CMML/CML. Complete remission (CR) was defined as a BM blast count of <5%. Minimal residual disease (MRD) status in both AML and MDS was assessed by high-sensitivity multiparameter flow cytometry.

### Study endpoints

The primary endpoint was disease relapse, defined as morphological recurrence of underlying malignancy after allo-SCT. Secondary endpoints included progression-free survival (PFS), defined as time from transplantation to relapse or death from any cause; non-relapse mortality (NRM), defined as death without prior relapse, with relapse considered as a competing risk; and the cumulative incidence of aGVHD (grades II-IV and III-IV) and chronic GVHD (cGVHD), defined and graded according to NIH consensus criteria [24, 25].

### Statistical considerations

The cumulative incidences of disease relapse, NRM, and GVHD were estimated, accounting for competing risks. Death before relapse; relapse or death with persistent disease; and death or relapse before GVHD were considered competing events for the respective three outcomes. In addition, patients with primary graft failure were excluded from the assessment of GVHD. Actuarial OS and PFS were estimated using the Kaplan-Meier method. Risk factors for disease relapse, NRM, and GVHD were evaluated in univariate and multivariate analyses using Fine-Gray regression to account for competing events. Predictors of OS and PFS were evaluated in univariate and multivariate analyses using Cox’s proportional hazards regression analysis. DPB1 subsets were forced in all multivariate models irrespective of statistical significance in univariate analysis. Among the remaining factors, those that were statistically significant in univariate analysis were considered in multivariate analysis. Backward elimination was used to determine the factors retained in the final multivariate model. Interaction effect by disease-risk was evaluated by performing separate analyses (stratifying) in the three disease-risk subgroups. In addition to DPB1, risk factors considered in the high-risk disease group included: diagnosis (AML vs MDS), recipient age (> vs ≤50 years), donor age (> vs ≤30 years), HCT-CI (> vs ≤3), donor/recipient gender, donor/recipient CMV status, conditioning intensity, and stem cell source. Disease status was evaluated based on remission status at transplant (CR1/CR2 vs other), MRD status (positive/negative), and the composite of disease status and MRD for patients in CR1/CR2; CR1/CR2 MRD-negative, CR1/CR2 MRD positive, CR1/CR2 MRD missing, not CR1/CR2. This grouping strategy was based on prior studies from our center and others demonstrating that patients transplanted in CRi or other incomplete response states have a significantly higher risk of relapse than those transplanted in CR [26–28]. Statistical significance was determined at the 0.05 level. Analyses were primarily conducted using STATA 18.0 (StataCorp LLC).

### Ethics approval and consent to participate

All methods were performed in accordance with the relevant guidelines and regulations. Approval has been obtained from MD Anderson Cancer Center Institutional Review Board (PA13-0774). The requirement for informed consent was waived by the ethics committee due to the retrospective nature of the study.

## Results

### Cohort and transplant features across DPB1 groups

A total of 541 patients were included in the analysis. DPB1 status was distributed as follows: DP-M (*n* = 176, 32%), DP-P (*n* = 219, 40%), DP-NP-GVH (*n* = 82, 15%), and DP-NP-HVG (*n* = 64, 12%) (Table 1). Overall, the median age of the recipients was 64 years (range, 18–77), with 41% aged 61–70 and 19% over the age of 70. The most common diagnoses were AML (53%) and MDS (23%), followed by MPN (14%), CMML (5%), and CML (4%).

**Table 1:** Baseline characteristics of 8/8 HLA-matched unrelated donor-recipient pairs receiving PTCy and MMF (*n* = 541).

	Overall *N* = 541	DP-M *N* = 176	DP-P *N* = 219	DP-NP-GvH *N* = 82	DP-NP-HvG *N* = 64
Recipient characteristics					
Recipient age					
Median (range), years	64 (18–77)	63 (18–77)	64 (20–77)	65 (26–77)	65 (30–76)
≤50 years	129 (24)	44 (25)	54 (25)	21 (26)	10 (16)
51–60 years	90 (17)	32 (18)	38 (17)	10 (12)	10 (16)
61–70 years	221 (41)	70 (40)	90 (41)	35 (43)	26 (41)
>70 years	101 (19)	30 (17)	37 (17)	16 (19)	18 (28)
Diagnosis					
AML	289 (53)	90 (51)	124 (57)	43 (52)	32 (50)
MDS	125 (23)	45 (26)	52 (24)	13 (16)	15 (23)
MPN/CMML/CML	127(24)	41 (23)	43 (20)	26 (32)	17 (27)
Disease risk category^a^					
High	192 (35)	57 (32)	87 (40)	28 (34)	20 (31)
Low/Intermediate	215 (40)	75 (43)	87 (40)	26 (32)	27 (42)
MPN/CMML/CML	127 (23)	41 (23)	43 (20)	26 (32)	17 (27)
Missing	7 (1)	3 (2)	2 (1)	2 (2)	0
Remission status					
CR1/CR2	258 (48)	81 (46)	108 (49)	40 (49)	29 (45)
Other	283 (52)	95 (54)	111 (51)	42 (51)	35 (55)
CR1/CR2/MRD-negative	95 (18)	30 (17)	43 (20)	12 (15)	10 (16)
CR1/CR2/MRD-positive	129 (24)	41 (23)	47 (21)	23 (28)	18 (28)
CR1/CR2/MRD missing	34 (6)	10 (6)	18 (8)	5 (6)	1 (2)
Disease beyond CR1/CR2	283 (52)	95 (54)	111 (51)	42 (51)	35 (55)
HCT-CI					
Median (range)	3 (0–11) [1,4]	3 (0–10) [1,4]	2 (0–10) [1,4]	3 (0–11) [1,5]	4 (0–10) [2,5]
Recipient CMV+					
N	372	122	159	54	37
Donor CMV−	243 (65)	78 (64)	100 (63)	38 (70)	27 (73)
Donor CMV+	129 (35)	44 (36)	59 (37)	16 (30)	10 (27)
Recipient CMV−					
*N*	167	53	59	28	27
Donor CMV−	103 (62)	31 (58)	43 (73)	15 (54)	14 (52)
Donor CMV+	64 (38)	22 (41)	16 (27)	13 (46)	13 (48)
Donor characteristics					
Donor age					
Median (range), years	29 (18–68)	27 (19–68)	29 (18–60)	33 (19–60)	33 (18–53)
≤25 years	161 (30)	64 (36)	67 (31)	15 (18)	15 (23)
26–30 years	140 (26)	50 (28)	58 (26)	21 (26)	11 (17)
31–35 years	89 (16)	23 (13)	42 (19)	11 (13)	13 (20)
>35 years	138 (25)	36 (20)	45 (20)	33 (40)	24 (37)
Donor/recipient sex					
F/F	83 (15)	27 (15)	39 (18)	10 (12)	7 (11)
F/M	85 (16)	25 (14)	34 (15)	13 (16)	13 (20)
M/F	131 (24)	53 (30)	46 (21)	20 (24)	12 (19)
M/M	237^a^ (44)	69 (39)	98 (45)	38 (46)	32 (50)
Missing	5 (1)	2 (1)	2 (1)	1 (1)	0
Transplant characteristics					
Conditioning regimen					
MAC	343 (63)	111 (63)	139 (63)	55 (67)	38 (59)
RIC	198 (37)	65 (37)	80 (36)	27 (33)	26 (41)
Stem cell source					
PB	472 (87)	146 (83)	196 (89)	74 (90)	56 (87)
BM	69 (13)	30 (17)	23 (10)	8 (10)	8 (12)
Maintenance therapy					
Yes	164 (30)	55 (31)	70 (32)	26 (32)	13 (20)
No	377 (70)	121 (69)	149 (68)	56 (68)	51 (80)
Transplant year					
Median (range) [IQRT]	2021 (2011–2024) [2020, 2023]	2021 (2011–2024)	2021 (2012–2024)	2021 (2013–2024)	2021 (2012–2024)
F/up in alive					
Median (range) [IQRT]	29 (3.5-125) [14,49]	32 (3.5-125)	29 (5-122)	22 (4-78)	25 (4-79)

^a^Risk classification by ELN for AML and r-IPSS for MDS.

*DP-M* DPB1 matched, *DP-P* DPB1 permissive mismatch, *DP-NP-GvH* DPB1 non-permissive mismatch graft-versus-host direction, *DP-NP-HvG* DPB1 non-permissive mismatch host-versus-graft direction, *AML* acute myeloid leukemia, *MDS* myelodysplastic syndromes, *MPN* myeloproliferative neoplasms, *CMML* chronic myelomonocytic leukemia, *CML* chronic myeloid leukemia, *CR* complete remission, *MRD* minimal residual disease, *HCT-CI* hematopoietic cell transplantation–specific comorbidity index, *CMV* cytomegalovirus, *MAC* myeloablative conditioning, *RIC* reduced-intensity conditioning, *PB* peripheral blood, *BM* bone marrow, *F* female, *M* male, *PTCy* post-transplant cyclophosphamide, *MMF* mycophenolate mofetil.

Disease-risk stratification using ELN for AML and IPSS-R for MDS classified 35% of patients as high risk and 40% as low/intermediate risk. Risk status could not be determined for 1% of the patients. At transplant, 48% were in CR1/CR2; and among these, MRD was detectable in 50% (*N* = 129), undetectable in 37% (*N* = 95), and unknown in 13% (*N* = 34). Pre-HCT disease status for remaining patients who were not in CR is seen in Supplementary Table 3. Given biologic and clinical heterogeneity, interpretation of DPB1 effects was focused on AML/MDS, while patients with MPN, CMML, or CML (23% of the cohort) were analyzed separately.

Donors had a median age of 29 years (range, 18–68), with 56% being 30 or younger. The most common donor-recipient sex combinations were male/male (M/M) (44%) and male/female (M/F) (24%). Cytomegalovirus (CMV) pairing was varied: among CMV-positive recipients (*n* = 372), 65% had CMV-negative donors, while among CMV-negative recipients (*n* = 167), 62% had CMV-negative donors.

MAC regimens were used in 63% of patients, while RIC regimens were used in 37%. The predominant stem cell source was PB in 87% of transplants, with BM used in 13%.

Baseline donor-recipient characteristics and transplant related variables including conditioning intensity and graft source were well-balanced across DPB1 groups (Table 1).

### Relapse: DP-P mismatch halves risk in high-risk AML/MDS

**Fig. 1:**
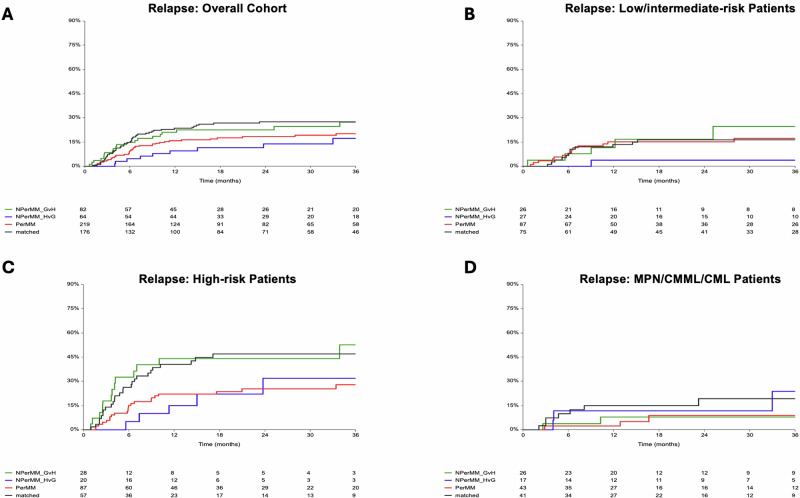
Two-year cumulative incidence of relapse post-transplant overall and stratified by disease risk. Disease relapse is shown for the overall cohort (**A**), low/intermediate-risk patients (**B**), high-risk AML/MDS patients (**C**), and MPN/CMML/CML patients (**D**). Results are shown according to patient DPB1 matching, including DPB1 matched (black), DPB1 permissive mismatch (red), DPB1 non-permissive mismatch graft-versus-host direction (green), and DPB1 non-permissive mismatch host-versus-graft direction (blue).

In the full cohort (*n* = 541), the 2-year cumulative incidence of relapse differed by DPB1 matching status: 28% with DP-M, 18% with DP-P, 22% with DP-NP-GVH, and 14% with DP-NP-HVG (Table 2 and Fig. 1A). Compared with DP-M, relapse risk was significantly reduced with DP-P (Hazard ratio (HR) 0.6, 95% confidence interval (CI) 0.4–0.9, *p* = 0.03), and with DP-NP-HVG (HR = 0.4, 95% CI 0.2–0.9, *p* = 0.02), but not with DP-NP-GVH (22%, HR 0.8, 95% CI 0.5–1.4, *p* = 0.5).

**Table 2:** Transplant outcomes across groups by DPB1 matching criteria^a^.

	*N* = 541	Relapse % (95% CI)	OS % (95% CI)	PFS % (95% CI)	TRM % (95% CI)	Grade 3–4 aGVHD by D180% (95% CI)	cGVHD % (95% CI)
**Overall (***n* = **541)**		**22 (18–25)**	**61 (56–65)**	**59 (55–64)**	**19 (16–22)**	**8 (6–10)**	**16 (13–20)**
DP-M	176	28 (22–35)	59 (51–66)	57 (49–64)	15 (10–21)	9 (5–14)	16 (12–23)
DP-P	219	18 (14–24)	62 (55–68)	60 (53–66)	22 (17–28)	8 (5–13)	16 (12–22)
DP-NP-GVH	82	22 (15–34)	56 (44–67)	58 (46–68)	19 (12–31)	5 (2–13)	10 (5–20)
DP-NP-HVG	64	14 (7–27)	69 (55–79)	68 (54–78)	18 (11–31)	6 (2–16)	22 (13–36)
**High-risk AML/MDS**	**192**	**35 (29–43)**	**48 (40–55)**	**45 (37–52)**	**20 (15–26)**	---	---
DP-M	57	47 (35–62)	43 (29–56)	39 (26–51)	14 (7–27)	---	---
DP-P	87	25 (17–37)	54 (42–64)	52 (40–62)	23 (15–34)	---	---
DP-NP-GVH	28	44 (29–67)	36 (18–54)	34 (17–52)	22 (11–44)	---	---
DP-NP-HVG	20	32 (15–69)	52 (25–74)	48 (22–70)	20 (8–48)	---	---
**Low/intermediate AML/MDS**	**215**	**14 (10–20)**	**68 (61–74)**	**68 (61–74)**	**18 (13–24)**	---	---
DP-M	75	16 (10–27)	67 (55–77)	66 (54–76)	18 (11–29)	---	---
DP-P	87	15 (9–25)	68 (57–77)	67 (56–76)	18 (11–28)	---	---
DP-NP-GVH	26	17 (7–41)	67 (41–83)	72 (49–86)	11 (4–33)	---	---
DP-NP-HVG	27	4 (0.5–25)	72 (50–86)	73 (51–86)	23 (11–47)	---	---
**MPN/CMML/CML**	**127**	**12 (7–20)**	**72 (62–79)**	**70 (60–77)**	**17 (11–26)**	---	---
DP-M	41	19 (10–38)	72 (54–84)	70 (51–82)	9 (3–26)	---	---
DP-P	43	9 (3–27)	68 (51–80)	61 (43–75)	30 (18–49)	---	---
DP-NP-GVH	26	8 (2–30)	72 (48–87)	74 (51–88)	18 (7–44)	---	---
DP-NP-HVG	17	12 (3–43)	80 (49–93)	81 (53–94)	7 (1–45)	---	---

^a^*OS* overall survival, *PFS* progression-free survival, *TRM* transplant-related mortality, *aGvHD* acute graft-versus-host disease, *cGvHD* chronic graft-versus-host disease, *AML* acute myeloid leukemia, *MDS* myelodysplastic syndromes, *MPN* myeloproliferative neoplasms, *CMML* chronic myelomonocytic leukemia, *CML* chronic myeloid leukemia, *DP-M* DPB1 matched, *DP-P* DPB1 permissive mismatch, *DP-NP-GvH* DPB1 non-permissive mismatch graft-versus-host direction, *DP-NP-HvG* DPB1 non-permissive mismatch host-versus-graft direction.

High-risk disease was the dominant adverse prognostic factor for relapse (HR: 2.7, 95% CI 1.8–4.2, *p* < 0.0001), OS (HR 1.9, 95% CI 1.4–2.6, *p* < 0.001), and PFS (HR 1.9, 95% CI 1.4–2.7, *p* < 0.0001) (Table 3). Outcomes were therefore stratified by disease risk classification (Supplementary Table 1).

**Table 3:** Comparison of outcomes by disease risk^a^.

2 years		Relapse		OS		PFS		TRM		6 m Grade 3-4 aGVHD		cGVHD	
	*N*	HR (95% CI)	*p*	HR (95% CI)	*p*	HR (95% CI)	*p*	HR (95% CI)	*p*	HR (95% CI)	*p*	HR (95% CI)	*p*
**AML/MDS**													
Low/intermediate-risk	215	Reference		Reference		Reference		Reference		Reference		Reference	
High-Risk	192	2.7 (1.8–4.2)	<0.001	1.9 (1.4–2.6)	<0.001	1.9 (1.4–2.7)	<0.0001	1.1 (0.7–1.8)	0.6	1.2 (0.6–2.5)	0.6	0.6 (0.4–1.2)	0.1
Missing	7	3.9 (1.1–14)	0.03	3.5 (1.5–8)	0.004	3.8 (1.6–8.7)	0.002	2.5 (0.9–7)	0.08	NE		1.0 (0.1–8)	0.9
**MPN/CMML/CML**	127	0.8 (0.4–1.5)	0.5	0.8 (0.5–1.3)	0.4	0.9 (0.6–1.3)	0.5	0.9 (0.5–1.5)	0.7	1.1 (0.5–2.5)	0.8	1.7 (0.9–2.8)	0.05

^a^*HR* hazard ratio, *CI* confidence interval, *OS* overall survival, *PFS* progression-free survival, *TRM* transplant-related mortality, *GVHD* graft versus host disease, *m* month, *AML* acute myeloid leukemia, *MDS* myelodysplastic syndromes, *MPN* myeloproliferative neoplasms, *CMML* chronic myelomonocytic leukemia, *CML* chronic myeloid leukemia, *NE* non-evaluable.

Among patients with low/intermediate-risk AML/MDS and MPN/CMML/CML, relapse incidence was uniformly low and comparable across DPB1 matching categories (Table 2 and Figs. 1B, D). All subsequent relapse and survival analyses were therefore limited to the high-risk AML/MDS group (*n* = 192), which accounted for most relapse events (Fig. 1C).

Within this high-risk AML/MDS cohort, DPB1 matching had a marked prognostic impact on relapse (Table 4 and Fig. 1C). Patients with DP-P donors experienced a 50% reduction in relapse rate compared to patients with DP-M donors (HR 0.5, 95% CI 0.3–0.8, *p* = 0.01). DP-NP-HVG mismatches showed a trend towards a possible protective effect (HR 0.4, 95% CI 0.2–1.1, *p* = 0.07), while DP-NP-GVH mismatches conferred no significant benefit (HR 1.03, 95% CI 0.5-2.0, *p* = 0.9). Strikingly, the 2-year relapse incidence among high-risk patients with DP-P mismatched donors approached that observed in low/intermediate-risk patients (Fig. 1B, D).

**Table 4:** Risk factors for relapse, overall survival, progression-free survival and transplant-related mortality in high-risk AML/MDS patients (*n* = 192)^a^.

	2-year relapse		2-year OS		2-year PFS		2-year TRM	
	HR (95% CI)	*p*	HR (95% CI)	*p*	HR (95% CI)	*p*	HR (95% CI)	*p*
**DP MM type**								
DP-M	1.0		1.0		1.0		1.0	
DP-P	0.5 (0.3–0.8)	0.01	0.8 (0.5–1.2)	0.3	0.7 (0.4–1.1)	0.1	1.6 (0.7–3.6)	0.3
DP-NP-GVH	1.03 (0.5–2)	0.9	1.4 (0.8–2.5)	0.3	1.4 (0.8–2.4)	0.3	1.6 (0.6–4.8)	0.4
DP-NP-HVG	0.4 (0.2–1.1)	0.07	0.7 (0.3–1.5)	0.3	0.7 (0.3–1.4)	0.3	1.5 (0.4–5.3)	0.5
**MDS vs AML**	1.6 (0.9–2.6)	0.09	1.02 (0.6–1.6)	0.9	1.1 (0.7–1.7)	0.8	0.5 (0.2–1.3)	0.2
**Remission status**								
CR1/CR2 MRD-negative	0.4 (0.2–0.96)	0.04	0.5 (0.3–0.9)	0.02	0.5 (0.2–0.8)	0.01	0.8 (0.3–1.9)	0.5
CR1/CR2 MRD-positive	0.8 (0.5–1.4)	0.4	0.7 (0.5–1.1)	0.2	0.8 (0.5–1.3)	0.4	1.05 (0.5–2.1)	0.9
CR1/CR2 MRD-missing	1.1 (0.4–3)	0.9	0.4 (0.1–1.3)	0.1	0.6 (0.2–1.6)	0.3	NE	
Not CR1/CR2	1.0		1.0		1.0		1.0	
**Recipient age**								
>50 vs ≤50			1.3 (0.8–2.2)	0.3	1.1 (0.7–1.8)	0.7	3.3 (0.9–11)	0.05
**HCT-CI, median**								
>3 vs ≤3	0.8 (0.5–1.3)	0.4	2.0 (1.4–3.1)	0.001	1.8 (1.2–2.7)	0.003	4.6 (2.2–9.4)	<.01
**Donor age** > **30 vs** **≤30**	1.5 (0.9–2.5)	0.1	1.02 (0.7–1.5)	0.9	1.1 (0.7–1.6)	0.7	0.6 (0.3–1.2)	0.1
**Donor/recipient sex**								
F/F	1.0		1.0		1.0		1.0	
F/M	0.6 (0.2–1.5)	0.3	1.0 (0.5–1.9)	0.9	1.1 (0.5–2)	0.9	1.9 (0.8–5.2)	0.2
M/F	0.8 (0.3–1.9)	0.6	0.8 (0.4–1.4)	0.4	0.8 (0.4–1.5)	0.5	0.9 (0.3–2.4)	0.8
M/M	0.9 (0.5–1.9)	0.9	0.6 (0.4–1.2)	0.2	0.7 (0.4–1.3)	0.3	0.5 (0.2–1.4)	0.2
Missing	1.6 (0.2–15)	0.7	0.8 (0.1–6.4)	0.9	0.8 (0.1–5.9)	0.8	NE	
**Recipient CMV**+								
Donor CMV−	1.0		1.0		1.0		1.0	
Donor CMV+	1.8 (0.9–3.3)	0.08	1.7 (1.05–3)	0.03	1.6 (0.9–2.5)	0.06	1.1 (0.5–2.4)	0.7
**Recipient CMV−**								
Donor CMV−	1.0		1.0		1.0		1.0	
Donor CMV+	1.3 (0.6–2.7)	0.6	0.7 (0.3–1.6)	0.4	0.9 (0.5–1.9)	0.9	0.5 (0.1–2.5)	0.4
**Conditioning regimen**								
MAC	0.9 (0.6–1.6)	0.9	0.9 (0.6–1.3)	0.5	0.9 (0.6-1.3)	0.5	0.7 (0.4–1.4)	0.4
RIC	1.0		1.0		1.0		1.0	
**Stem cell source**								
PB	0.8 (0.4–1.5)	0.4	1.2 (0.6–2.3)	0.5	1.02 (0.6–1.9)	0.9	1.6 (0.5–5)	0.4
BM	1.0		1.0		1.0		1.0	

^a^*HR* hazard ratio, *CI* confidence interval, *OS* overall survival, *PFS* progression-free survival, *TRM* transplant-related mortality, *AML* acute myeloid leukemia, *MDS* myelodysplastic syndromes, *CR* complete remission, *MRD* measurable residual disease, *HCT-CI* hematopoietic cell transplantation–specific comorbidity index, *MAC* myeloablative conditioning, *RIC* reduced-intensity conditioning, *PB* peripheral blood, *BM* bone marrow, *F* female, *M* male, *CMV* cytomegalovirus, *NE* not estimable.

Remission and MRD status were also prognostic factors for relapse (Table 4). Considered as a composite (remission/MRD) covariate, patients transplanted in CR1/CR2 with MRD negativity had the lowest relapse risk (HR 0.4, 95% CI 0.2–0.96, *p* = 0.04), whereas MRD-positive patients transplanted in CR1/CR2 had relapse incidence comparable to those transplanted beyond CR1/CR2 (HR 0.8, 95% CI 0.5–1.4, *p* = 0.4). None of the additional factors considered reached statistical significance in univariate analysis.

Multivariate analysis within the high-risk AML/MDS cohort yielded consistent results. After adjusting for remission/MRD status, DP-P mismatch remained independently protective, reducing relapse risk by half (HR 0.50 (95% CI 0.3–0.8, *p* = 0.01(Table 5). In this analysis, MRD status further stratified risk: patients in MRD negative CR had the lowest relapse risk (HR 0.4 (95% CI 0.2–1.0), *p* = 0.05). In contrast, MRD-positive patients transplanted in CR1/CR2 had relapse incidences similar to those transplanted beyond CR1/CR2 (HR 0.8, 95% CI 0.5–1.4, *p* = 0.5).

**Table 5:** Multivariate analysis for transplant outcomes in high-risk AML/MDS patients^a^.

	Relapse		OS		PFS		TRM	
	HR (95% CI)	*p*	HR (95% CI)	*p*	HR (95% CI)	*p*	HR (95% CI)	*p*
**DP-P**	0.5 (0.3–0.8)	0.01	0.8 (0.5–1.2)	0.3	0.7 (0.4–1.1)	0.1	1.7 (0.7–3.8)	0.2
**DP-NP-GVH**	1.0 (0.5–2.1)	0.9	1.4 (0.7–2.5)	0.3	1.3 (0.7–2.3)	0.4	1.8 (0.6–5.1)	0.3
**DP-NP-HVG**	0.4 (0.2–1.1)	0.08	0.7 (0.3–1.4)	0.3	0.6 (0.3–1.2)	0.2	1.5 (0.4–5.2)	0.5
**CR1/CR2 MRD-negative**	0.4 (0.2–1.0)	0.05	--		0.5 (0.3–0.9)	0.03	--	
**CR1/CR2 MRD-positive**	0.8 (0.5–1.4)	0.5	--		0.8 (0.5–1.2)	0.3	--	
**CR1/CR2 MRD-missing**	1.1 (0.4–3.2)	0.9	--		0.5 (0.2–1.5)	0.2	--	
**HCT-CI** > **3**	--		2.2 (1.4–3.2)	<0.001	1.7 (1.1–2.5)	0.01	4.6 (2.2–9.6)	<0.001
**Donor/** **Recipient CMV** **Reactive/** **Reactive**	--		1.8 (1.1–2.8)	0.01	--		--	

Factors (in addition to DPB1) considered in multivariate analysis included:

Remission/MRD status for relapse; remission/MRD status, HCT-CI, and donor/recipient CMV for OS; remission/MRD status and HCT-CI for PFS, and HCT-CI and recipient age for TRM.

^a^DPB1 subsets were forced in all multivariate models.

*HR* hazard ratio, *CI* confidence interval, *PFS* progression-free survival, *OS* overall survival, *TRM* treatment-related mortality, *CR* complete remission, *MRD* measurable residual disease, *HCT-CI* hematopoietic cell transplantation–specific comorbidity index, *DP* HLA-DPB1.

### Progression-free and overall survival: trends favor DP-P without statistical significance

Overall, two-year OS and PFS rates were not significantly different across DPB1 categories. OS estimates were 59% for DP-M, 62% for DP-P, 56% for DP-NP-GVH, and 69% for DP-NP-HvG; corresponding PFS estimates were 57%, 60%, 58%, and 68%, respectively (Table 2). For OS, compared to DP-M, hazard ratios were 0.9 (95% CI 0.7–1.3, *p* = 0.9) for DP-P, 1.1 (95% CI 0.7–1.7, *p* = 0.6) for DP-NP-GVH, and 0.7 (95% CI 0.4–1.2, *p* = 0.2) for DP-NP-HvG. For PFS, compared to DP-M, hazard ratios were 0.9 (95% CI 0.7–1.3, *p* = 0.7) for DP-P, 1.04 (95% CI 0.7–1.6, *p* = 0.8) for DP-NP-GVH, and 0.7 (95% CI 0.4–1.1, *p* = 0.1) for DP-NP-HvG.

**Fig. 2:**
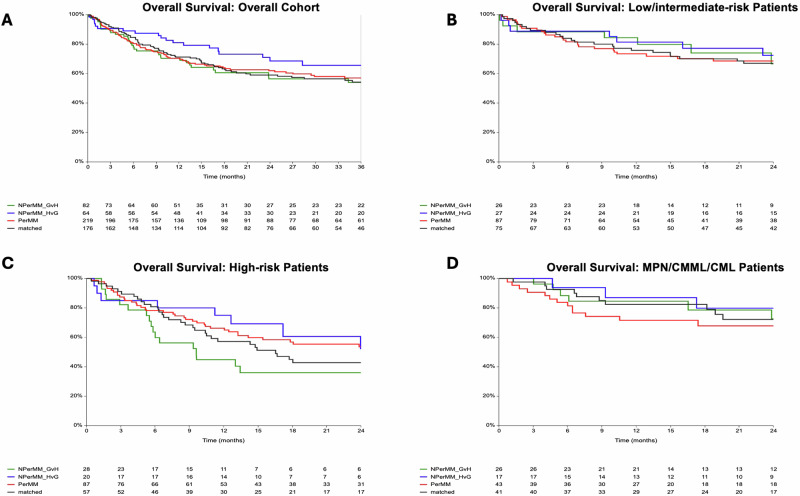
Two-year overall survival (OS) in the overall cohort and by disease risk. Kaplan–Meier curves display OS in the overall cohort (**A**), in low/intermediate-risk patients (**B**), in high-risk patients (**C**), and in MPN/CMML/CML patients (**D**). Results are shown according to patient DPB1 matching, including DPB1 matched (black), DPB1 permissive mismatch (red), DPB1 non-permissive mismatch graft-versus-host direction (green), and DPB1 non-permissive mismatch host-versus-graft direction (blue).

**Fig. 3:**
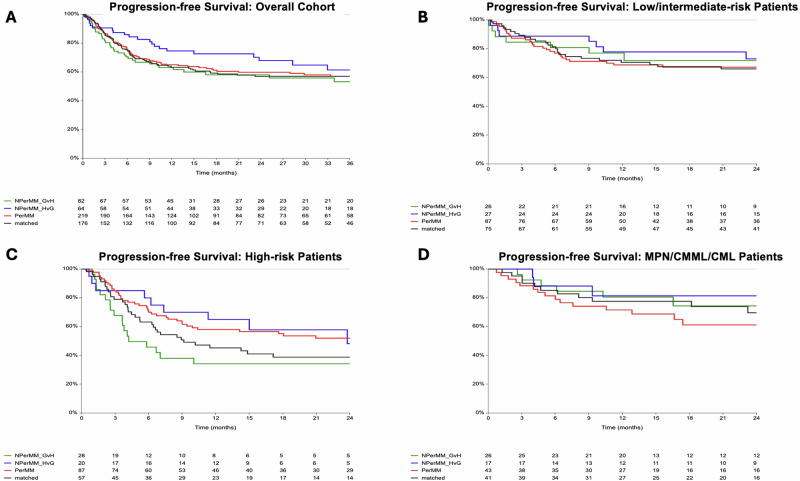
Two-year progression-free survival (PFS) in the overall cohort and by disease risk. Kaplan–Meier curves display PFS in the overall cohort (**A**), in low/intermediate-risk patients (**B**), in high-risk patients (**C**), and in MPN/CMML/CML patients (**D**). Results are shown according to patient DPB1 matching, including DPB1 matched (black), DPB1 permissive mismatch (red), DPB1 non-permissive mismatch graft-versus-host direction (green), and DPB1 non-permissive mismatch host-versus-graft direction (blue).

Despite the relapse benefit, DP-P did not translate into significantly better survival in high-risk AML/MDS. Two-year OS was higher with DP-P and DP-NP-HVG pairs (54% and 52%, respectively) compared DP-M pairs (43%), though these differences did not reach statistical significance (DP-P vs DP-M: HR 0.8, 95% CI 0.5–1.2, *p* = 0.3; DP-MM-HVG vs DP-M: HR 0.7, 95% CI 0.3–1.5, *p* = 0.3) (Fig. 2A). Similarly, PFS was highest in DP-P pairs (52%) compared with others (39%), suggesting a favorable trend that did not reach statistical significance (HR 0.7, 95% CI 0.5–1.03, *p* = 0.07) (Fig. 3A). Among low-risk AML/MDS and MPN/CMML/CML groups, 2-year OS and PFS estimates were uniformly favorable and comparable across DP matching categories (Table 2, Figs. 2 and 3). Significant predictors of OS and PFS in univariate analysis in high-risk AML/MDS included remission/MRD status and HCT-CI. In addition, donor CMV status in CMV positive recipients was associated with OS but not PFS (Table 4).

Multivariate analyses for OS and PFS were performed only in the high-risk AML/MDS to investigate the independent effect of DP matching (Table 5). DPB1 mismatch did not independently affect OS or PFS. Indeed, transplantation in CR1/CR2 with MRD-negativity demonstrated improvement in PFS (HR 0.50, 95% CI 0.3–0.9, *p* = 0.03) and trended towards OS improvement (HR 0.55, 95% CI 0.30–1.03, *p* = 0.062).

Hematopoietic Cell Transplantation-Comorbidity Index (HCT-CI) score >3 was also associated with inferior OS (HR 2.2, 95% CI 1.4–3.2, *p* < .0001) and PFS (HR 1.7, 95% CI 1.15–2.5, *p* = 0.01). In addition, CMV reactive patient and donor pairs had significantly inferior OS (HR 1.8, 95% CI 1.1–2.8, *p* = 0.01).

### Non-related mortality: age and comorbidity as dominant predictors

At 2-years post-transplant, non-relapse mortality (NRM) was 19% overall, with incidences of 15% for DP-M, 22% for DP-P, 19% for DP-NP-GVH, and 18% for DP-NP-HvG (Table 2 and Supplementary Fig. 2). The trend toward higher TRM with DP-P versus DP-M did not reach statistical significance (HR 1.6, 95% CI 0.9–2.5, *p* = 0.07) in univariate analysis.

Since DP-P was associated with reduced relapse risk in high-risk AML/MDS, we evaluated risk factors for TRM in this subset (Table 4). In this analysis, DPB1 matching did not independently influence TRM, but HCT-CI scores >3 (HR 4.6, 95% CI 2.2–9.4, *p* < 0.01) were strongly associated, and recipient age >50 years tended (HR 3.3, 95% CI 0.9–11, *p* = 0.05) to be associated with higher TRM rate. In multivariate analysis adjusting for HCT-CI and recipient age, only HCT-CI remained significant (HR 4.6, 95% CI 2.2–9.6, *p* < 0.001) predictors of TRM.

### GVHD: determined by classical risk factors, not DPB1

**Fig. 4:**
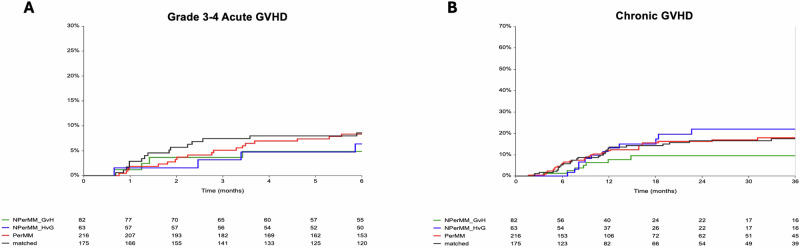
Cumulative incidence of acute and chronic GVHD by DPB1 matching status. **A** Cumulative incidence of grade III–IV acute GVHD within 6 months post-transplant. **B** Cumulative incidence of chronic GVHD up to 36 months post-transplant. Results are shown according to patient DPB1 matching, including DPB1 matched (black), DPB1 permissive mismatch (red), DPB1 non-permissive mismatch graft-versus-host direction (green), and DPB1 non-permissive mismatch host-versus-graft direction (blue).

DPB1 matching did not affect the incidence of aGVHD or cGVHD. By day 180 post-transplant, the cumulative incidence of grade III-IV aGVHD was 8%, with no significant differences by DPB1 matching status when compared with DP-M (DP-P: HR 0.96, 95% CI 0.5–1.9, *p* = 0.9; DP-NP-GVH: HR 0.5, 95% CI 0.2–1.7, *p* = 0.3; DP-NP-HVG: HR 0.7, 95% CI 0.4–2.2, *p* = 0.2, Supplementary Table 2). At 2 years post-transplant, the cumulative incidence of cGVHD was 16% overall, again without association to DPB1 status (Table 2 and Fig. 4A, B). Of note, given the relatively low incidence of grade III-IV and cGVHD, analyses were not stratified by disease risk. In univariate analysis, donor gender and conditioning intensity were the only factors associated with III-IV aGVHD, whereas conditioning intensity, stem cell source, MPN/CMML/CML diagnosis, and HCT-CI were the only factors associated with cGVHD.

On multivariate analysis, female donor sex was associated with increased rate of grade III-IV aGVHD (HR 2.2, 95% CI 1.2–4.1, *p* = 0.01) (Table 6). Paradoxically, MAC was associated with a reduced rate of grade III-IV aGVHD (HR 0.4, 95% CI 0.2–0.8, *p* = 0.006) but an increased rate of cGVHD (HR 2.0, 95% CI 1.1–3.6, *p* = 0.02). PB grafts increased cGVHD risk (HR 3.6, 95% CI 1.1–12.2, *p* = 0.03). Patients with MPN/CMML/CML also had a higher risk of cGVHD compared those with AML/MDS (HR 1.6, 95% CI 1.02–2.7, *p* = 0.04).

**Table 6:** Multivariable analysis of risk factors for GVHD (overall population)^a^.

	Grade III-IV aGVHD at day 180		cGVHD at 2 years	
Risk factor	HR (95% CI)	*p*	HR (95%CI)	*p*
DP-P	0.9 (0.4–1.8)	0.8	0.9 (0.5–1.5)	0.7
DP-NP-GVH	0.6 (0.2–1.8)	0.3	0.5 (0.2–1.1)	0.07
DP-NP-HVG	0.7 (0.2–1.9)	0.5	1.2 (0.6–2.4)	0.5
Female donor	2.2 (1.2–4.1)	0.01	----	
MAC vs RIC regimen	0.4 (0.2–0.8)	0.006	2.0 (1.1–3.6)	0.02
PB vs BM	----		3.6 (1.1–12.2)	0.03
HCTCI > 3 vs ≤ 3	----		0.6 (0.4–0.9)	0.05
MPD vs AML/MDS (as part of risk assessment)	----		1.6 (1.02–2.7)	0.04

^a^*aGVHD* acute graft-versus-host disease, *AML* acute myeloid leukemia, *BM* bone marrow, *cGVHD* chronic graft-versus-host disease, *CI* confidence interval, *DPB1* HLA-DPB1, *GVH* graft-versus-host, *HCT-CI* Hematopoietic Cell Transplantation–Comorbidity Index, *HR* hazard ratio, *HVG* host-versus-graft, *MAC* myeloablative conditioning, *MDS* myelodysplastic syndrome, *MPD* myeloproliferative disorder, *PB* peripheral blood, *RIC* reduced-intensity conditioning.

Factors (in addition to DPB1) considered in multivariate analysis included:

Donor gender and conditioning intensity for III-IV aGVHD, conditioning intensity, stem cell source, MPD/CMML/CML diagnosis, and HCT-CI for cGVHD.

In summary, in patients with high-risk AML/MDS, DP-P substantially reduced relapse rates but did not significantly improve OS or PFS. This is likely due to competing TRM driven by age and comorbidity burden in these patients. In low/intermediate-risk AML/MDS and MPN/CMML/CML, outcomes were favorable, and DPB1 status had limited impact on patient outcomes.

## Discussion

In this contemporary, homogenous cohort of 8/8 HLA-matched unrelated donor SCT in myeloid malignancies using PTCy-based GVHD prophylaxis, DPB1-permissive mismatching was associated with a meaningful reduction in disease relapse, without increasing aGVHD, cGVHD, or TRM. Importantly, the protective effect of permissive mismatching in reducing relapse risk was most evident in the high-risk AML/MDS subgroup, whereas outcomes in low-risk AML/MDS and MPN/CMML/CML were largely comparable across all DPB1 matching categories. These findings highlight the dominant influence of disease biology on long-term results. The magnitude of effect on relapse in the high-risk group was clinically relevant: permissive mismatching conferred a 50% relative reduction in relapse risk (HR 0.50, 95% CI 0.3–0.8), corresponding to an absolute reduction in 2-year relapse risk of 25% with DP-P pairs. Notably, relapse incidences in high-risk AML/MDS patients with DP-P pairs approached those of the lower-risk groups, suggesting that permissive mismatching attenuates the impact of high-risk genetics and the adverse effect of uncontrolled disease (including CR with MRD positivity, or beyond CR1/CR2 at time of transplant). Although the relapse benefit did not translate into statistically significant improvements in PFS or OS in this dataset, it is plausible that with longer-term follow up, the early relapse prevention afforded by permissive mismatching may result in a meaningful improvement in PFS, as suggested by the trend observed in this dataset (DP-P vs others, HR for PFS 0.7, 95% CI 0.5–1.03, *p* = 0.07).

This observation is particularly relevant in the current landscape of allo-SCT. Despite considerable advances in conditioning regimens, supportive care, and GVHD prophylaxis, relapse remains the major cause of treatment failure after transplant [1, 2]. Among interventions tested, only two have consistently achieved large and clinically meaningful effects. The BMTCTN1901 trial by Scott et al. [15] demonstrated that intensification of conditioning (MAC) reduced relapse in high-risk AML by almost 30% when compared with RIC (47.3% vs 67.8% at 18 months, *p* < 0.01), albeit at the cost of higher TRM. Similarly, tyrosine kinase inhibitors (TKI) targeting FLT3 have consistently shown clear benefit with 20–30% absolute risk reduction (HR ~ 0.25–0.40) in randomized studies [29–31]. By contrast, other approaches, including maintenance with hypomethylating agents, have produced only modest and inconsistent effects [16, 32–35]. A randomized clinical trial with subcutaneous azacitidine maintenance failed to show improved PFS or OS [17], and retrospective analyses reported only marginal benefit, with 3-year progression rates of 29% vs 33% (*p* = 0.09) [20]. These benefits, while statistically notable in selected settings, remain modest compared with improvements observed after MAC or FLT3-TKI maintenance and have not been consistent across trials. More recently, sequential approaches, including fractionation of busulfan therapy [20] and use of NK-based immunotherapies [19], have shown some promise in reducing relapse, although these remain investigational.

Biologically, DPB1-permissive mismatching supports the concept that carefully controlled class II alloimmunity can amplify GVL effects. HLA-DP is often retained on myeloid blasts, but downregulation of the HLA class II transactivator can silence HLA-DR, -DQ, and -DP, enabling leukemic escape from donor CD4+ cells [36]. DPB1 polymorphisms define TCE groups that shape the peptide-binding groove and its interactions with the T-cell receptor. In permissive DPB1 mismatches, donor and recipient alleles within the same TCE group maintain structural similarity and present largely overlapping peptide repertoires despite allelic mismatching, resulting in intermediate-avidity CD4+ responses that can enhance CD8+ cytotoxicity, however, do not provoke excessive tissue injury [37, 38]. This balanced alloreactivity broadens the post-transplant T-cell repertoire beyond self-restricted tumor antigens to include direct recognition of the mismatched DP heterodimer, thereby overcoming antigen loss and HLA class I downregulation used by adverse-risk myeloid clones to evade immunity [37, 39]. In contrast, non-permissive DPB1 mismatches involve alleles from more divergent peptide repertoires, generating a substantially broader alloantigen landscape, including both direct and indirect recognition of the mismatched HLA, with much greater potential for alloreactivity, tissue injury, and GVHD [37, 38]. Conversely, DP-matched grafts lack this additional allo-antigenic axis, leaving donor CD4⁺ T cells largely dependent on minor histocompatibility antigens, which may be insufficient to control genetically complex, immune-evasive AML/MDS (Supplementary Fig. 3).

In this study, permissive mismatching in the PTCy era improved relapse without increasing GVHD or TRM. This contrasts with pre-PTCy observations, where permissive DPB1 mismatches decreased relapse risk, but increased aGVHD [3, 6, 37]. PTCy appeared to abrogate this risk by modulating alloreactive integrations responsible for the balance between GVH and GVL through selective depletion or disablement of rapidly proliferating alloreactive T cells, while preserving regulatory T-cell populations [37, 40–42]. Our findings support the notion that in the PTCy era, permissive DPB1 mismatching uniquely preserves or enhances GVL effects without increasing GVHD. This represents a novel, practical, and readily implementable approach capable of delivering relapse protection of similar magnitude to MAC and FLT3-TKI maintenance, but without the toxicity of intensified conditioning or the uncertainty of pharmacologic therapy.

Our findings differ from the recent European registry analysis by Arrieta-Bolanos et al., in which permissive DPB1 mismatching was not associated with reduced relapse among recipients of 10/10 matched unrelated donors receiving PTCy. Whilst the overall registry cohort was large, the 10/10 PTCy subgroup was substantially smaller with relatively few relapse events (*n* = 254), resulting in wide confidence intervals and limiting power to detect clinically meaningful effects. In addition, this cohort was heterogenous with respect to underlying diseases, disease-risk, conditioning regimens, and transplant practices, which may dilute signals confined to biologically high risk populations. In contrast, our analysis focused on a more selected North American high-risk AML/MDS population, where differences in disease management, timing to transplantation, and donor selection practices may render donor-related effects more detectable. Together, these considerations suggest that DPB1-associated relapse effects may be context-specific and highlight the value of focused, disease-specific analyses.

These results have direct implications for donor selection. In high-risk AML/MDS, particularly in patients transplanted with residual disease, prioritizing a DPB1-permissive donor is justifiable, given the magnitude of relapse reduction observed without excess toxicity. The effect size is greater, if not at least comparable, to many post-transplant maintenance strategies, with the advantage that it is realized up-front in the donor selection process. Thus, DPB1 permissive mismatching is a promising, implementable strategy to improve transplant outcomes in high-risk AML/MDS patients. Moreover, considering that disease morphology and MRD status remain integral predictive factors of overall relapse risk, DPB1 permissiveness should be weighed alongside disease biology, MRD status, comorbidity, and logistical factors.

Limitations of this study include its retrospective design, potential for unmeasured confounders, limited sample size in smaller DPB1 subgroups (particularly HvG mismatches), and limited follow-up time, which may be insufficient to capture long-term survival effects. Furthermore, we did not incorporate TCE-core models or allele directionality, which may further refine permissive assignments [12]. Ultimately, external validation of these results using large registry-based datasets, including TCE-based DPB1 modelling and MRD stratification, is required.

## Conclusion

In summary, DPB1-permissive mismatching is an actionable strategy to reduce relapse in high-risk AML/MDS in the PTCy era, without incurring additional GVHD or NRM. In our cohort, permissive mismatching conferred a 25% absolute risk reduction at 2-years, suggesting preservation of GVL effects without excess alloreactivity and narrowing the outcome gap between high- and low/intermediate-risk disease. Future work should prospectively validate these findings, integrate MRD-guided strategies, and evaluate whether TCE-Core models further enrich donor selection using PTCy.

## Supplementary information

Supplementary Material

## Data Availability

The underlying data presented in this study are available upon request to the corresponding author.
