# Supplementary material for: Reduced relapse in high risk acute myeloid leukemia and myelodysplastic neoplasms with permissive HLA-DPB1 mismatches and post-transplant cyclophosphamide
Source: Leukemia. 2026 Mar 9;40(5):934–45. doi: 10.1038/s41375-026-02907-4 (PMC13149028; doi:10.1038/s41375-026-02907-4)

Supplementary Table 1: Baseline characteristics of 8/8 HLA-matched unrelated donor-recipient pairs receiving PTCy and MMF by disease risk (n=541).[1]

Characteristic	Overall N=541	Low/intermediate risk N=215	High-risk N=192	MPD/CMML/ CML N=127
**DPB1 matching**				
DP-M	176 (32)	75 (35)	57 (30)	41 (32)
DP-P	219 (40)	87 (40)	87 (45)	43 (34)
DP-GVH MM	82 (15)	26 (12)	28 (15)	26 (20)
DP-HvG MM	64 (12)	27 (12)	20 (10)	17 (13)
**Recipient age**				
Median (range)	64 (18-77) [52, 69]	62 (20-77)	65 (18-77)	64 (32-77)
≤50	129 (24)	60 (28)	41 (21)	27 (21)
>50-60	90 (17)	34 (16)	30 (16)	26 (20)
>60-70	221 (41)	78 (36)	81 (42)	58 (46)
>70	101 (19)	43 (20)	4 (21)	16 (13)
**Recipient CMV+**				
N	372 (69)	151 (70)	135 (70)	80 (63)
Donor CMV-	243 (65)	101 (67)	88 (65)	51 (64)
Donor CMV+	129 (35)	50 (33)	47 (35)	29 (36)
**Recipient CMV-**				
N	167 (31)	63 (29)	57 (30)	46 (36)
Donor CMV-	103 (62)	39 (62)	35 (61)	29 (63)
Donor CMV+	64 (38)	24 (38)	22 (39)	17 (37)
**Diagnosis**				
AML	289 (53)	146 (68)	143 (75)	0
MDS	116 (21)	69 (23)	49 (25)	78 (61)
MPD/CMML/CML	136 (24)	0	0	49 (39)
AML/MDS combined	442 (82)	215 (100)	192 (100)	28 (22)
CML/MPD combined	99 (18)	0	0	99 (78)
**Remission Status**				
CR1/CR2	258 (48)	129 (60)	108 (56)	21 (16)
Beyond CR1/CR2	283 (52)	86 (40)	84 (44)	106 (83)
CR1/CR2/MRD-negative	95 (18)	58 (27)	36 (19)	1 (0.8)
CR1/CR2/MRD-positive	129 (24)	62 (29)	63 (33)	4 (3)
CR1/CR2/MRD missing	34 (6)	9 (4)	9 (5)	16 (13)
**HCT-CI**				
Median (range)	3 (0-11) [1,4]	3 (0-11)	3 (0-10)	3 (0-10)
0-1	166 (31)	70 (33)	59 (31)	34 (27)
2	82 (15)	31 (14)	28 (15)	21 (16)
3	96 (18)	42 (19)	27 (14)	27 (21)

[1] **Abbreviations:** AML, acute myeloid leukemia; BM, bone marrow; CMML, chronic myelomonocytic leukemia; CMV, cytomegalovirus; CML, chronic myeloid leukemia; CR, complete remission; DPB1, HLA-DPB1; F, female; GVH, graft-versus-host; HVG, host-versus-graft; HCT-CI, Hematopoietic Cell Transplantation–Comorbidity Index; IQR, interquartile range; M, male; MAC, myeloablative conditioning; MDS, myelodysplastic syndrome; MMF, mycophenolate mofetil; MPD, myeloproliferative disorder; MRD, measurable residual disease; PB, peripheral blood; PTCy, post-transplant cyclophosphamide; RIC, reduced-intensity conditioning.

4	74 (14)	38 (18)	18 (9)	18 (14)
>4	123 (23)	34 (16)	60 (31)	27 (21)
**Donor Characteristics**				
**Donor age**				
median (range)	29 (18-68)	29 (19-55)	29 (18-68)	28 (18-56)
<=25	161 (30)	56 (26)	58 (30)	45 (35)
>25-30	140 (26)	58 (27)	54 (28)	27 (21)
>30-35	89 (16)	38 (18)	27 (14)	24 (19)
>35	138 (25)	56 (26)	49 (25)	30 (24)
Missing	13 (2)	7 (3)	4 (2)	1 (1)
**Donor/recipient sex**				
F/F	83 (15)	28 (13)	28 (15)	26 (10)
F/M	85 (16)	35 (16)	30 (16)	19 (15)
M/F	131 (24)	49 (23)	47 (24)	32 (25)
M/M	237 (44)	100 (46)	85 (44)	50 (39)
Missing	5 (1)	3 (1)	2 (1)	0
**Transplant Characteristics**				
**Conditioning regimen**				
MAC	343 (63)	118 (55)	116 (60)	103 (81)
RIC	198 (37)	97 (45)	76 (40)	24 (19)
**Stem cell source**				
PB	472 (87)	178 (83)	170 (88)	117 (92)
BM	69 (13)	37 (17)	22 (11)	10 (8)
**Maintenance therapy**				
Yes	164 (30)	71 (33)	72 (37)	20 (16)
No	377 (70)	144 (67)	120 (62)	107 (84)
**TP year**				
Median (range) [IQRT]	2021 (2011-2024) [2020, 2023]	2021 (2012-2024)	2021 (2012-2024)	2021 (2011-2024)
**F/up in alive**				
Median (range) [IQRT]	29 (3.5-125) [14,49]	31 (8-125)	28 (4-80)	25 (3-122)

Supplementary Table 2: Risk factors for GVHD in the overall population (n=541).[2]

	Grade II-IV aGVHD (6m)		Grade III-IV aGVHD (6m)		Chronic GVHD (6m)	
**Risk factor**	**HR (95%CI)**	**p**	**HR (95%CI)**	**p**	**HR (95%CI)**	**p**
**DP MM type**						
DP-M	1.0		1.0		1.0	
DP-P	0.8 (0.6-1.2)	0.4	0.96 (0.5-1.9)	0.9	0.98 (0.6-1.6)	0.9
DP-NP-HVG	1.1 (0.7-1.7)	0.7	0.5 (0.2-1.7)	0.3	0.5 (0.2-1.3)	0.2
DP-NP-GVH	0.7 (0.4-1.1)	0.1	0.7 (0.2-2.2)	0.6	1.2 (0.6-2.4)	0.5
Matched vs all other	1.1 (0.8-1.5)	0.4	1.2 (0.6-2.3)	0.5	1.1 (0.7-1.7)	0.7
**Disease risk**						
Low/intermediate risk	1.0		1.0		1.0	
High risk	0.9 (0.7-1.3)	0.6	1.2 (0.6-2.5)	0.6	0.6 (0.4-1.2)	0.1
MPD/CMML/CML	0.9 (0.6-1.3)	0.6	1.1 (0.5-2.5)	0.8	1.7 (0.9-2.8)	0.05
Missing	0.7 (0.2-3.2)	0.7	NE		1.0 (0.1-7.9)	0.9
**Disease status**						
CR1/CR2	1.2 (0.9-1.6)	0.2	0.8 (0.4-1.5)	0.6	0.7 (0.5-1.1)	0.2
Beyond CR1/CR2	1.0		1.0		1.0	
**Recipient age**						
>50 vs <=50	1.04 (0.7-1.5)	0.8	2.3 (0.9-5.9)	0.08	1.1 (0.6-1.8)	0.8
**HCT-CI**						
>3 vs <=3	1.3 (0.9-1.8)	0.05	1.5 (0.8-2.9)	0.2	0.6 (0.3-0.9)	0.04
**Donor age**						
>30 vs <=30	1.03 (0.8-1.4)	0.8	0.8 (0.4-1.5)	0.5	1.3 (0.8-1.9)	0.3
**Donor/recipient sex**						
F/F	1.0		1.0		1.0	
F/M	0.7 (0.4-1.1)	0.1	0.8 (0.3-1.9)	0.6	0.6 (0.4-2.1)	0.9
M/F	0.7 (0.4-1.1)	0.09	0.4 (0.1-0.9)	0.05	1.1 (0.5-2.2)	0.8
M/M	0.7 (0.4-.9)	0.05	0.4 (0.2-0.9)	0.03	0.7 (0.4-1.4)	0.3
Donor F	1.2 (0.9-1.6)	0.2	2.2 (1.2-4)	0.01	1.2 (0.7-1.9)	0.5
F/F vs. others	1.5 (1.02-2.1)	0.04	2.1 (1.1-4.3)	0.03	1.1 (0.6-2.1)	0.6
**Recipient CMV+**						
Donor CMV-	1.0		1.0		1.0	
Donor CMV+	1.3 (0.9-1.8)	0.1	0.9 (0.5-1.9)	0.9	1.4 (0.8-2.5)	0.2
**Recipient CMV-**						
Donor CMV-	1.0		1.0		1.0	
Donor CMV+	1.8 (0.9-3.3)	0.06	0.2 (0.03-1.8)	0.2	0.9 (0.4-1.9)	0.8
**Donor CMV+**	1.4 (1.04-1.8)	0.03	0.7 (0.4-1.4)	0.4	1.2 (0.8-1.9)	0.3
**Conditioning regimen**						
MAC	1.1 (0.8-1.5)	0.4	0.4 (0.2-0.8)	0.008	2.6 (1.5-4.5)	0.001
RIC	1.0		1.0		1.0	

[2] **Abbreviations:** aGVHD, acute graft-versus-host disease; BM, bone marrow; cGVHD, chronic graft-versus-host disease; CI, confidence interval; CMML, chronic myelomonocytic leukemia; CMV, cytomegalovirus; CML, chronic myeloid leukemia; CR, complete remission; DPB1, HLA-DPB1; F, female; GVH, graft-versus-host; GVHD, graft-versus-host disease; HCT-CI, Hematopoietic Cell Transplantation–Comorbidity Index; HR, hazard ratio; HvG, host-versus-graft; M, male; MAC, myeloablative conditioning; MDS, myelodysplastic syndrome; MPD, myeloproliferative disorder; PB, peripheral blood; PTCy, post-transplant cyclophosphamide; RIC, reduced-intensity conditioning.

**Stem cell source**						
PB	1.1 (0.7-1.7)	0.6	1.02 (0.4-2.6)	0.9	4.1 (1.3-13)	0.02
BM	1.0		1.0		1.0	

Supplementary Table 3: Distribution of high risk AML and MDS pre-HCT responses in patients not in CR1/CR2 at time of transplant[3]

**Response prior to SCT High-risk AML or MDS**	**AML** N 143 (%)	**MDS** N 49 (%)
**CR1/CR2**	98 (69)	10 (20)
**Not CR1/CR2**	45 (31)	39 (80)
MLFS	14 (10)	N/A
CRi	10 (7)	N/A
Untreated	2 (1)	N/A
No response *(active disease)*	19 (13)	18 (37)
Hematologic response	N/A	16 (33)
Marrow CR	N/A	5 (10)

[3] **Abbreviations:** AML, acute myeloid leukemia; CR, complete remission; CRi, complete remission with incomplete count recovery; MDS, myelodysplastic syndrome; MLFS, morphologic leukemia-free state

Supplementary Table 4: Maintenance therapy by DPB1 status[4]

	Overall		High-risk AML/MDS	
**DPB1**	N	N (%) maintenance	N	N (%) maintenance
**DP-M**	176	55 (31)	57	22 (39)
**DP-P**	219	70 (32)	87	32 (37)
**DP-NP-GVH**	82	26 (32)	28	12 (43)
**DP-NP-HVG**	64	13 (20)	20	6 (30)
P value		*0.3*		*0.8*

[4] **Abbreviations:** AML, acute myeloid leukemia; DPB1, HLA-DPB1; DP-M, DPB1 matched; DP-P, DPB1 permissive mismatch; DP-NP-GvH, DPB1 non-permissive mismatch graft-versus-host direction; DP-NP-HvG, DPB1 non-permissive mismatch host-versus-graft direction, MDS, myelodysplastic syndrome;

Supplementary Figure 1: Classification of HLA-DPB1 T-cell epitope (TCE) groups and mismatch categories. A) Common DPB1 alleles and their assignment to TCE3 and TCE4 groups B) Donor-recipient pairings stratified by TCE groups. Mismatches are categorized as permissive or non-permissive, with directionality as graft-versus-host (GvH) and host-versus-graft (HvG).

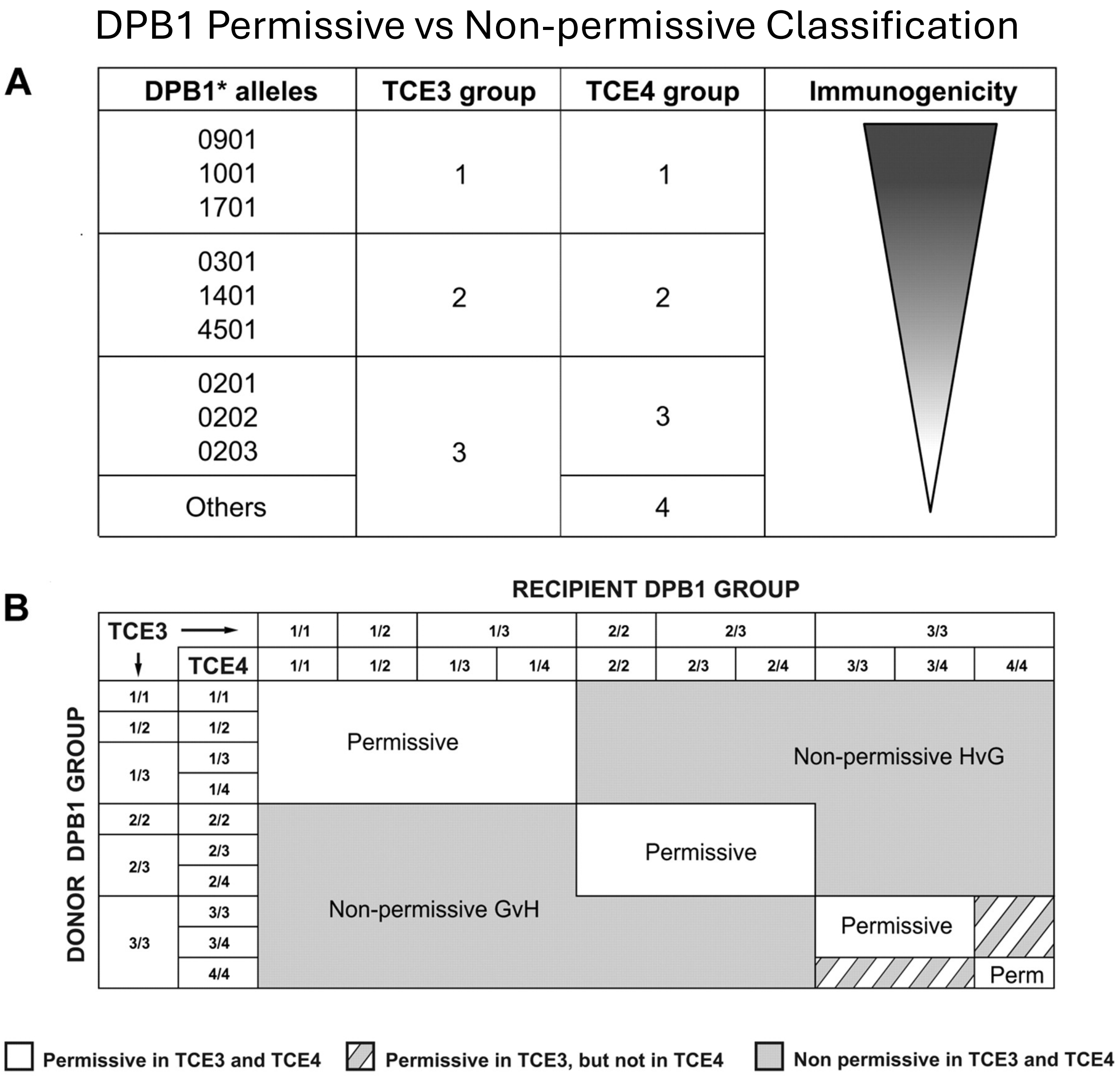

Supplementary Figure 2: Two-year non-relapse mortality (NRM) in the overall cohort and by disease risk. Kaplan–Meier curves display NRM in the overall cohort (A), in low/intermediate-risk patients (B), in high-risk patients (C), and in MPN/CMML/CML patients (D). Results are shown according to patient DPB1 matching, including DPB1 matched (black), DPB1 permissive mismatch (red), DPB1 non-permissive mismatch graft-versus-host direction (green), and DPB1 non-permissive mismatch host-versus-graft direction (blue).

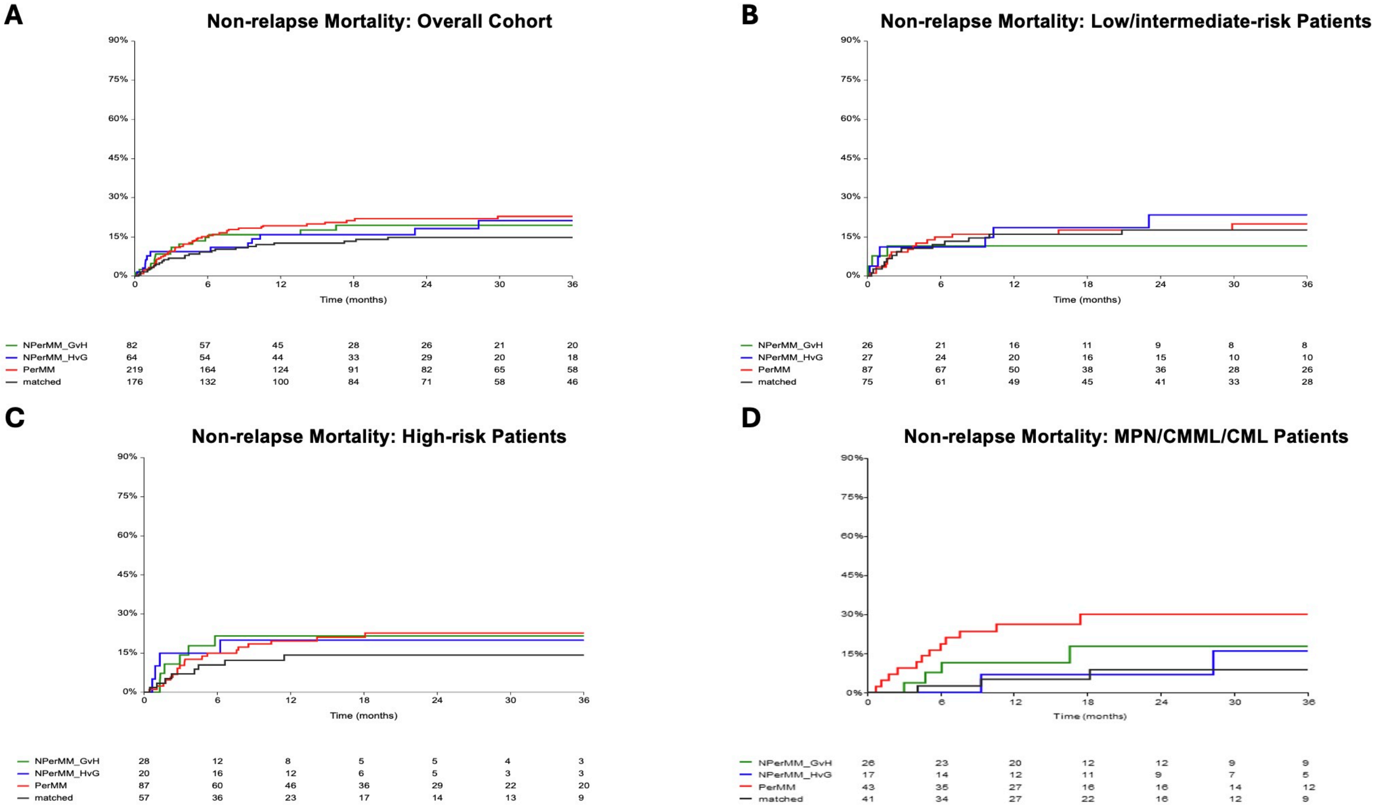

Supplementary Figure 3: Proposed biologic rationale for DPB1-permissive mismatches and effect on relapse.

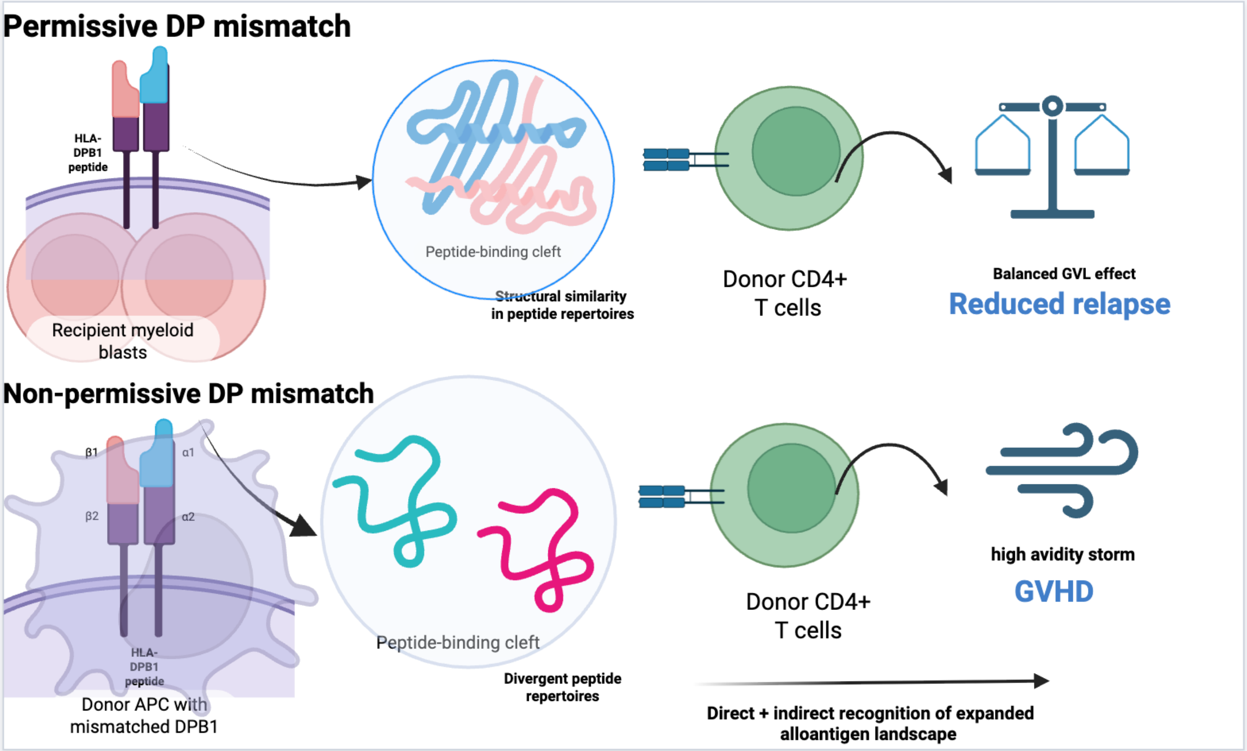

Supplement: Supplementary file 1 — Supplementary Material [file 41375_2026_2907_MOESM1_ESM.pdf]
